# Evaluating the implementation of the active life improving health behavior change program “BCP-VAMOS” in primary health care: Protocol of a pragmatic randomized controlled trial using the RE-AIM and CFIR frameworks

**DOI:** 10.3389/fpubh.2022.726021

**Published:** 2022-09-12

**Authors:** Lisandra Maria Konrad, Cezar Grontowski Ribeiro, Elaine Cristina Maciel, Camila Tomicki, Fabiana Almeida Brito, Fabio Araujo Almeida, Tânia Rosane Bertoldo Benedetti

**Affiliations:** ^1^Research Center on Physical Activity and Health, Sports Center, Federal University of Santa Catarina, Florianopolis, Santa Catarina, Brazil; ^2^Department of Physical Education, Federal Institute of Parana, Palmas, Parana, Brazil; ^3^Department of Health Promotion, College of Public Health, University of Nebraska Medical Center, Omaha, NE, United States

**Keywords:** Implementation Science, protocol study, program evaluation, public health, care innovation

## Abstract

**Introduction:**

The effective translation of evidence-based interventions has contributed to implementing actions that impact public policies and the population's health. However, there is a gap in the literature regarding the factors associated with the successful implementation of these interventions. The Active Life Improving Health Behavior Change Program (BCP-VAMOS) uses behavioral strategies to promote an active and healthy lifestyle in the community. Characterized as a health innovation, it also provides health professionals with online training to implement the program in Primary Health Care (PHC). Our study describes a pragmatic trial that aims to evaluate the implementation of BCP-VAMOS, version 3.0, in PHC in southern Brazil.

**Methods and analysis:**

A pragmatic randomized controlled trial (PRCT) of two arms comparing a group of PHC professionals who will participate in a traditional didactic approach (control group) vs. a group that will receive ongoing support (intervention group) for the implementation of BCP-VAMOS. The intervention will be available to adults (≥18 years old) registered at PHC. Program recipient's will be assessed at baseline and post-intervention (9 months after) to measure markers of physical activity and eating behavior (primary outcomes). Program's implementation process will be monitored for 12 months and will be evaluated using the RE-AIM and Consolidated Framework for Implementation Research (CFIR) frameworks.

**Discussions:**

The survey findings can be used widely throughout Brazil, guiding the work of health professionals, service planners and policy-makers. Also, the results may help to inform the national health promotion policy to plan interventions and improve the implementation of programs in PHC. This research results will provide practical guidance for researchers to develop similar protocols to implement and adapt public health interventions.

**Ethics and dissemination:**

Ethics approval has been granted by the Human Research Ethics Committee of the Federal University of Santa Catarina (UFSC), Brazil, under no. 1394492. Results will be published in full as open access in the UFSC library and main trial results and associated papers in high-impact peer-reviewed journals.

**Trial registration number:**

RBR-2vw77q—Brazilian Registry of Clinical Trials – ReBEC (http://www.ensaiosclinicos.gov.br).

## Introduction

Since the publication of global strategies to promote physical activity, healthy eating, and risk prevention associated with lifestyle behaviors (1), evidence-based practices (EBPs) have contributed to the implementation of public health interventions (2). In this context, a concern has arisen regarding whether these interventions are implemented as intended, and are able to promote the expected impact on the population's health (3).

Many dissemination and implementation (D&I) models, theories, and frameworks (4, 5) are available to help understanding this process. However, there is a gap in the literature concerning the implementation and adaptation of EBPs in certain contexts (6, 7). Practical implications usually include the utilization of strategies to increase adoption rates among health professionals and adapt protocols to local needs (7, 8). Nevertheless, despite the notable advance in Implementation Science (IS), the complexity that characterizes translational processes is sometimes ignored, limiting the understanding of the real effects of interventions (9).

The implementation facilitation strategies, as they deal with facing barriers to the adoption of EBPs, assist in the translational process, identifying how practices and providers can adopt and maintain interventions (4, 10, 11). The most used facilitation method includes providing didactic training for health professionals with protocols and tools to implement an intervention (12, 13). However, this strategy has been insufficient to promote widespread adoption and ineffective when it comes to the success of the implementation (11, 13). Combining traditional didactic approaches with ongoing support seems to improve the effect size of the strategies used in the intervention (14, 15). Adopting the action planning model as ongoing support after the training has effectively changed behavior, as it takes barriers and contingencies into account during the implementation of innovative practice in the health area (16).

Ongoing support improves the provider's adherence to EBPs and the quality of health services implementation. Nevertheless, further research is needed to identify effective strategies and how ongoing support affects the process and results of implementation (15). This research proposes to evaluate the impact of online training with ongoing support centered on the action planning approach and to compare it with the traditional didactic approach in the implementation of an intervention.

One way of understanding the mechanisms involved in implementation is the use of pragmatic trials with random assignment in “real world” settings (9, 17). They are designed precisely to inform decision-makers in practice (2), to report what actually works in a particular context (18), and to provide results concerning the feasibility and sustainability of interventions (19).

In light of this, we suggest a pragmatic trial to evaluate the implementation of the Behavior Change Program called Active Life Improving Health (BCP-VAMOS), which aims to motivate adults and elderly living in the community to adopt an active and healthy lifestyle (20). Online training will be available to health professionals as an adaptation strategy to assist in the implementation of the protocol (21).

The trial will be supported by RE-AIM (Reach, Effectiveness, Adoption, Implementation, Maintenance) (22) and CFIR (Consolidated Framework for Implementation Research) (23) to understand the factors involved in the implementation. Both frameworks are based on theories related to the adoption, implementation, and maintenance of EBPs (24, 25). Their combined use enables to measure success and to evaluate the factors that explain and improve implementation outcomes (26). Therefore, this study aims to describe the design and methods of a pragmatic trial that intends to evaluate the implementation of BCP-VAMOS, version 3.0, in Primary Health Care (PHC).

## Methods and analysis

### Study design

**Figure 1:**
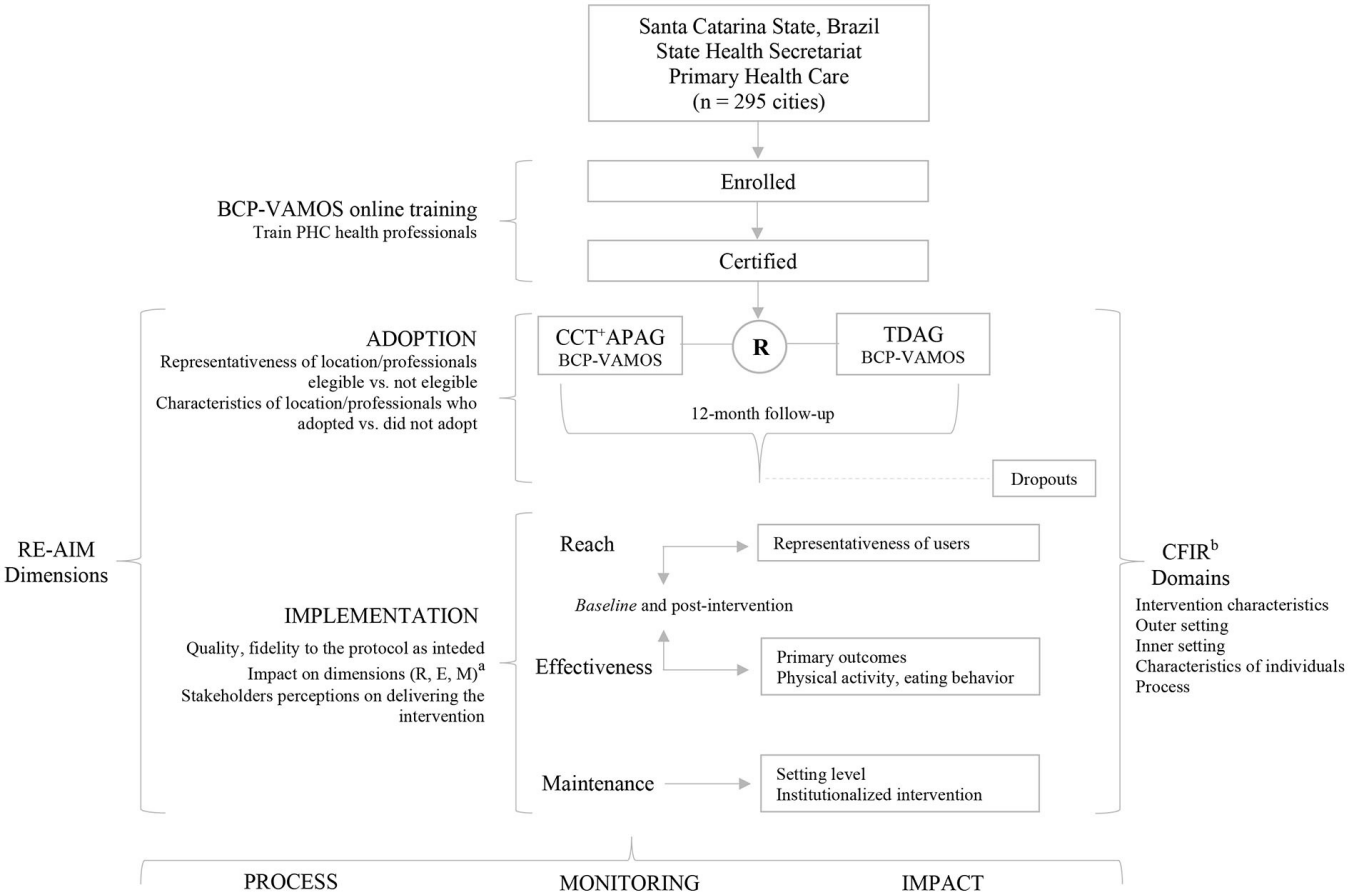
Flow chart of procedures of the pragmatic randomized controlled trial (PRPT) for the implementation of the Active Life Improving Health Behavior Change Program (BCP-VAMOS), version 3.0, in Primary Health Care, Brazil. PHC, Primary Health Care; R, randomization; CCT+APAG, consultee-centered training plus action planning approach group; TDAG, traditional didactic approach group. ^a^The reach (R), effectiveness (E), and maintenance (M) dimensions will be considered to evaluate the impact in the implementation process between the two groups, CCT^+^APA vs. TDA. ^b^Consolidated Framework for Implementation Research.

A pragmatic randomized controlled trial (PRCT) will be carried out to evaluate the implementation of BCP-VAMOS, version 3.0, in PHC (see Figure 1). The design was classified by the PRECIS-II tool (PRagmatic-Explanatory Continuum Indicator Summary) (27) and described following the SPIRIT 13 checklist (Standard Protocol Items: Recommendations for Interventional Trial), as it is the description of a study protocol that provides health outcomes (28). This research is part of the community-based trial entitled “VAMOS Program—Active Life Improving Health.” It was approved by the Human Research Ethics Committee of the Federal University of Santa Catarina, Brazil, under no. 1394492, and registered in ReBEC—Brazilian Registry of Clinical Trials (http://www.ensaiosclinicos.gov.br/) using the RBR-2VW77Q indicator on 20 July 2019. Any changes to the protocol will be registered in ReBEC.

### Description of BCP-VAMOS

**Figure 2:**
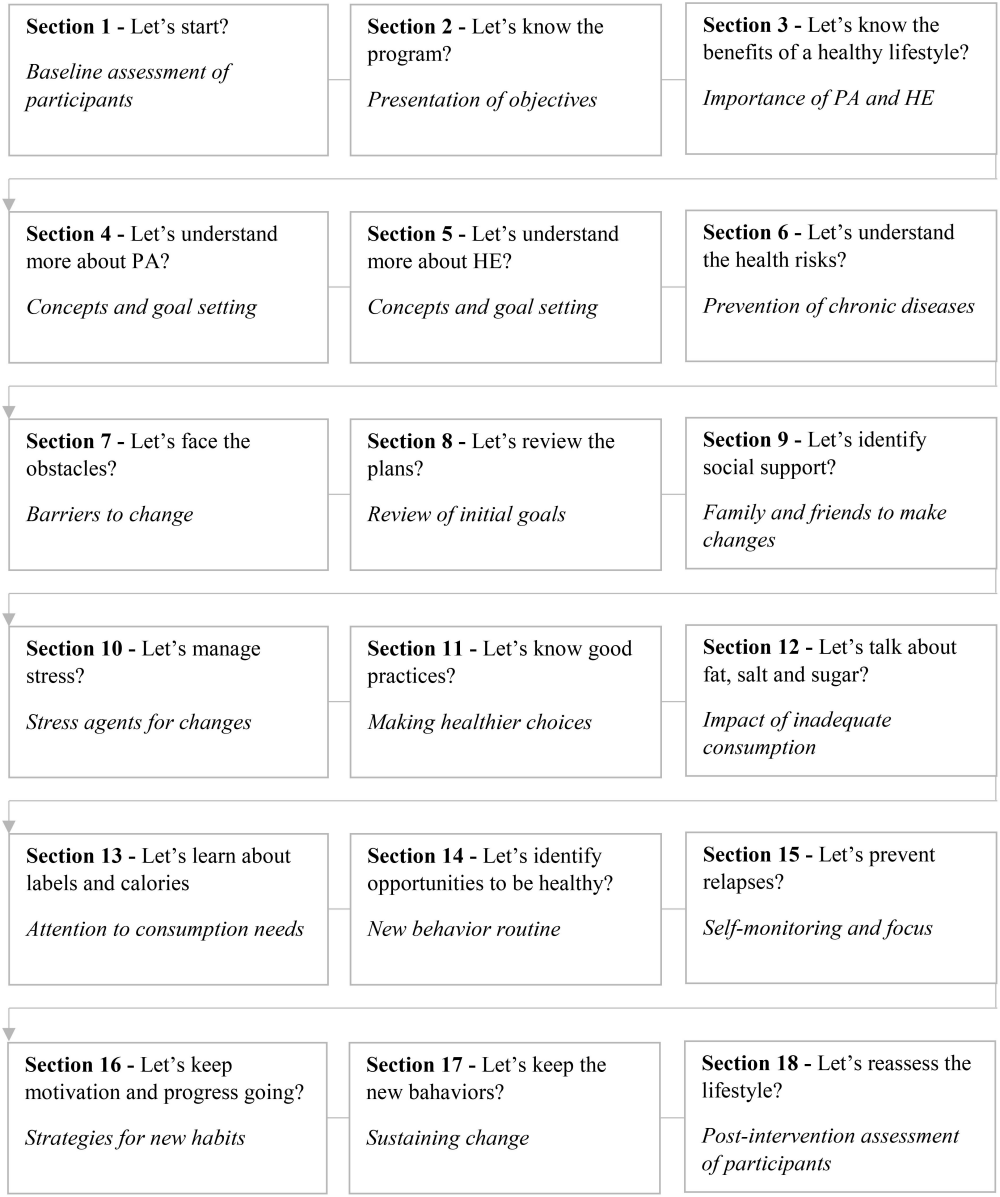
Sections and themes of BCP-VAMOS, version 3.0. Brazil. Adapted from Benedetti et al. (29). The didactic material is free of charge and is distributed to participants in each of the sections. PA, physical activity; HE, healthy eating.

Active Life Improving Health (VAMOS) is a behavior change program that aims to motivate adults and elderly (18 or older) individuals to adopt an active and healthy lifestyle through physical activity and healthy eating (20). In the current version (3.0), the face-to-face modality is developed to be implemented over 9 months in 18 sections. These sections are scheduled to be delivered weekly, biweekly and/or monthly. Each section is taught with a duration of up to 2 h, conducted by a PHC professional whose methodology is health education. The didactic material consists of 18 printed notebooks that address topics related to the benefits of physical activity and healthy eating, in addition to the development of strategies to effect and maintain new life habits (see Figure 2) (29).

### Study location and participants

The study will be conducted with professionals from health teams that work in PHC in the state of Santa Catarina, southern Brazil. The state has 295 municipalities and an estimated population of 7,250,000 inhabitants. It is the third state in the national human development index (HDI = 0.774), has the lowest social inequality index (GINI = 0.494) and the highest performance index of the unique health system in the country (IDSUS = 6–6.99). PHC in Santa Catarina is divided into seven regions, with 1,824 primary care centers, 1,855 family health teams, 234 primary care teams (doctor, nurse, dentist), and 301 multidisciplinary teams (nutritionist, pharmacist, physiotherapist, physical education professional, psychologist, social worker). These teams take into consideration the multi-professional health care policy and are formed by 7,056 PHC professionals with bachelor's degree (30). As the VAMOS program in the face-to-face version requires a minimum of 8 people to start a group and we expect that around 45 health professionals can complete the training, a total of at least 360 adults may be recruited to participate in the program.

### Description of the study design

The study design is based on strategies recommended by experts (4, 31, 32), centered on organizational support (14), to assist in the fidelity of the implementation and delivery of the BCP-VAMOS. Online training was developed, and educational material was prepared in electronic and printed forms to be distributed, free of charge, to health professionals and program participants. In addition, ongoing support will be structured based on information about facilitation (problem-solving and barriers) and adaptability (fidelity) of the implementation protocol (14, 16). This support will be delivered by a specialist researcher at the PHC. Therefore, PHC professionals will be allocated into two groups to assess the impact of the program's implementation: (1) control group (CG), with PHC professionals who will participate in a traditional didactic approach without ongoing support, and (2) intervention group (IG), with consultee-centered training plus action planning approach.


$$\begin{array}{l} \begin{array}{l} CG=Traditional\;Didactic\;Approach\;(TDA) \end{array} \end{array}$$

**Figure 3:**
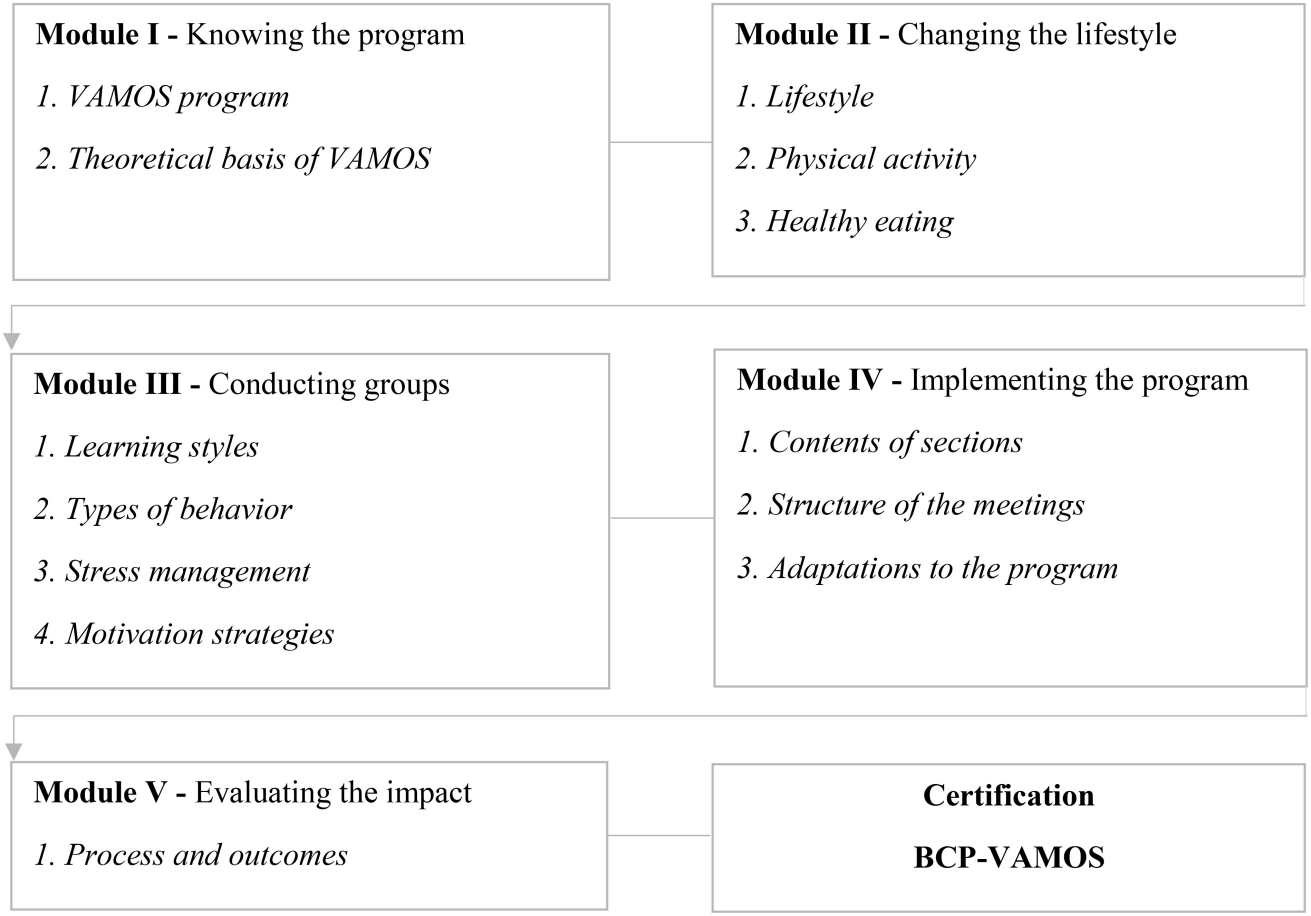
Online training learning modules for implementation of BCP-VAMOS, version 3.0. Brazil. Adapted from Konrad et al. (21).

This group will attend a 20-h online training (see Figure 3) and receive teaching materials with information and content related to the program's implementation protocol, recruitment, retention, and evaluation of participants (Table 1). The online training follows a didactic model with self-instructional language and will be made available free of charge in the e-learning modality, with the purpose to train PHC professionals (called facilitators) to plan, conduct and evaluate the program in different contexts. The online training has five modules that include themes and contents divided into 13 chapters. The content is so that the PHC professional learns about the program's proposal, the concepts and markers for behavior change, strategies for managing groups of people, the structure and content for implementation, and finally, the program's evaluation forms (see Figure 3) (21). PHC professionals must present a performance of at least 80% in the final evaluation—It is a 30-question multiple-choice quiz—to be certified as a BCP-VAMOS facilitator. The online training was developed by the BCP-VAMOS researchers' team and validated by an expert panel composed of researchers from related fields and PHC professionals (21).


$$\begin{array}{l} IG=Consultee-Centered\;Training\\ \;Plus\;Action\;Planning\;Approach\;(CCT+APA) \end{array}$$

**Table 1:** Description of strategies for groups with and without ongoing support for the implementation of BCP-VAMOS, version 3.0. Brazil.

**Intervention stage**	**Traditional didactic approach group^a^**	**Consultee-centered training + action planning approach group^b^**
**Program dissemination, participants enrollment**	Printed material to publicize and recruit the community: posters, flyers, and business cards.	Printed material plus guidelines to optimize dissemination and recruitment: who, where, when, and how.
**Program sections** (18 sections)	T-shirt with program logo for the provider. Digital guidelines, printed material to conduct the sections, record the frequency, and monitor and evaluate the sections.	T-shirt, manual, printed material plus guidelines for each of the sections: preparation of the site, reception, content guiding, participation registration, and individual section monitoring.
**Participants evaluation** (baseline and post-intervention)	Digital guidelines, video, and printed material to record the participants' baseline and post-intervention assessment data.	Digital guidelines, video, printed material plus guidelines for carrying out evaluations about: location, professionals involved, and the necessary care for reliable measures.

^a^20-h online training (21) + teaching materials. ^b^20-h online training (21)+ teaching materials + structured messages throughout the implementation process.

This group will participate in the 20-h online training as described above and will receive ongoing support centered on the provider, using an action planning strategy. Ongoing support (see Table 1) will be structured by a specialist PHC researcher and provided during 12 months for all the implementation stages: recruiting the community, conducting program sessions, and evaluating participants. Support will focus on the process of identifying barriers to program implementation, selecting strategies to deal with barriers and goals for recruiting and retaining new participants. Before the start of each stage, the intervention provider will receive a message reinforcing the strategy that should be used. After each stage, another contact will be made to assess the used strategy and provide new feedback. Support will be delivered through interviews with the provider using a smartphone messaging app.

Both groups will be monitored simultaneously. If the CG providers have questions during the implementation process, these will be answered at their origin, but, being careful not to offer strategies similar like those IG will be offered. Thus, both groups will be monitored by one single researcher to avoid communication bias and to ensure that information from the IG is not passed on to the CG. This researcher will be a specialist in the health area, particularly in PHC, and received training to implement the BCP-VAMOS.

### Recruitment and eligibility

The online training will be disclosed to PHC professionals electronically and the enrolment will be performed on the BCP-VAMOS website (vamos.ufsc.br). The training will be available during 4 months for qualification and certification of health professionals. When the PHC professionals are enrolled in the online training, they will be informed about the research objectives and procedures. Their participation will be accepted after they sign the Free and Informed Consent Form.

#### Inclusion criteria

The PHC professional must have a college degree, work in PHC, and be authorized by the manager of the municipal health department to implement the BCP-VAMOS.

#### Exclusion criteria

Those enrolled PHC professionals who do not complete the online training will be excluded from the study.

### Randomization

The randomization and allocation of health professionals into the two groups (CCT + APA vs. TDA) will follow a sequence randomly generated by a computer. This process will be carried out after all the PHC professionals complete the online training. Health professionals from the same workplace will be assigned to the same group should more than one professional participate in the online training. After randomization, they will be asked to implement the BCP-VAMOS in their workplaces. Randomization and group assignment will be under the responsibility of one single researcher and will be blinded to the others involved in the implementation strategies and data collection.

### New description

The randomization and allocation of health professionals into the two groups (CCT + APA vs. TDA) will be performed after all PHC professionals complete the online training. Through a randomly generated computer sequence, we will randomize each facility to one of the intervention arms with all eligible professionals within each facility randomized to the same intervention arm to avoid cross-contamination within the same facility. However, multiple facilities within the same city may be randomized to different groups. After randomization, they will be asked to implement BCP-VAMOS in their workplaces. The randomization and assignment of groups will be under the responsibility of a single researcher and will be blind to the others involved in the implementation strategies and data collection.

### Outcomes evaluation

The evaluation of the BCP-VAMOS implementation process will be carried out based on RE-AIM (22) and CFIR (23). These frameworks enable to measure the quantitative and qualitative impact of an intervention on public health (26).

This study, despite focusing exclusively on the characteristics of the implementation, will describe the individual dimensions of RE-AIM (Reach = R and Effectiveness = E) to determine the impact of implementation and ongoing support on the program adaptation to the real world. The organizational dimensions (Adoption = A, Implementation = I, Maintenance = M) will be assessed to determine the factors involved in the implementation's success (see Table 2).

**Table 2:** RE-AIM and CFIR information to evaluate the implementation of BCP-VAMOS, version 3.0. Brazil.

**RE-AIM, dimensions**	**RPT aims and outcomes measures**
^**a**^**Reach:** measure of participation in the intervention (individual level)	**Aim:** to describe the number, proportion, and representativeness of the participants in the intervention. **Quantitative:** recruitment, participation, and retention rates of participants calculated by PHC professional who will deliver the intervention. *Data collection:* registration of interested, enrolled, retained, and dropouts.
^**a**^**Effectiveness:** measure of the intervention's impact (individual level)	**Aim:** to show the results of the primary intervention outcomes. **Quantitative:** proportion of participants showing a behavior change in PA and EB by PHC professional. *Data collection:* baseline and post-intervention assessment.
**Adoption:** measure of the participation of location/teams interested in initiating the intervention (staff level)	**Aim:** to describe number, proportion, representativeness, and the characteristics of the location/PHC professionals who initiated the delivery of the intervention. **Quantitative:** adoption rate, demographic and social data of location/PHC professionals vs. the eligible ones. *Data collection:* electronic form filled out by PHC professionals. **Qualitative:** reasons that influenced the adoption and barriers to not adopt the intervention. *Data collection:* interview with PHC professionals.
**Implementation:** degree to which the intervention was delivered as intended	**Aim:** to evaluate the quality of the implementation, fidelity of delivery of the intervention, and the degree to which the strategies are implemented as intended. **Quantitative:** proportion of location/PHC professional that implemented the intervention as intended. *Data collection:* settings visit and process observation. **Qualitative:** protocol adaptations, barriers, and facilitators for implementation. *Data collection:* interview with managers, PHC professionals, and participants.
**Maintenance:** measure the extent to which the intervention is institutionalized (setting level)	**Aim:** to verify the continuity of the intervention by location/PHC professionals. **Quantitative:** number of new groups that have been implemented over time or after the delivery of the intervention by a PHC professional. **Qualitative:** reasons for implementing new groups or discontinuity. *Data collection:* interview with managers and PHC professionals.
**CFIR, domains**
**Intervention characteristics:** main attributes that influence the success of the intervention	**Aim:** to describe the characteristics that influence the successful implementation of the intervention. **Qualitative:** adaptability, complexity of implementation, barriers, and facilitators. *Data collection:* interview with managers, PHC professionals, and participants.
**Outer setting**: external factors of the organization can have positive or negative influences on the implementation of the intervention	**Aim:** to identify external factors that may influence the successful implementation of the intervention. **Qualitative:** perception of the patient's needs and resources for implementation. *Data collection:* interview with managers and PHC professionals.
**Inner setting**: aspects of the organization's functioning and structure that influence the success of the intervention	**Aim:** to describe the internal characteristics of the organization. **Qualitative:** climate and readiness of location/PHC professionals for implementation of the intervention. *Data collection:* interview with managers and PHC professionals.
**Characteristics of individuals**: level of knowledge, belief, attitude, and engagement of providers during implementation that may determine the success of the intervention	**Aim:** to identify personal characteristics that may influence the implementation of the intervention. **Qualitative:** knowledge and beliefs, self-efficacy, identification with the organization, and other personal attributes. *Data collection:* interview with PHC professionals.
**Process:** decision making of the organization's agents that influence the success of the intervention	**Aim:** to assess the organization's commitment to the intervention protocol. **Qualitative:** involvement of location/PHC professionals who led the intervention, barriers, and facilitators for implementation. *Data collection:* monitoring the stages and interviewing managers, PHC professionals, and participants.

PRCT, pragmatic randomized controlled trial; PA, physical activity; EB, eating behavior. ^a^These RE-AIM will be used to assess the impact of the implementation on the intervention group and control group, whose data will be collected by the PHC professionals (providers) of the intervention.

**Reach** is a measure at the individual level of participation, which refers to the characteristics and percentages of the target population's participation in the intervention (22). In this study, reach will be assessed as the number, proportion, and representativeness of program recipients enrolled and retained in the program. We will assess the involvement of program recipients based on three reach rates: recruitment (ratio between the number of users interested in participating in the program in relation to the number of exposed users—users served at the UBS during the period when the program was announced, multiplied by 100); participation (ratio between the number of participants who will start the program in relation to the number of eligible participants in the program, multiplied by 100); retention (ratio between the number of participants who will complete and the number of participants who started the program, multiplied by 100). These reach rates will be calculated by PHC professional, comparing the IG to the CG.

To reach the participants (recruitment), we will make available printed materials (posters, folders) and we will suggest the use of social networks, in addition to the media (radio, television, internet) to publicize the program. We recommend employing these strategies during the 30-day period prior to the start of the program. In order to recruit participants, we will establish the following eligibility criteria: being 18 years of age or older, being registered and having been attended at UBS in the 30 days prior to the start of the program (30 days from the disclosure), not meeting the minimum recommendations of PA practice (150 min per week of moderate and/or vigorous PA) and/or having inappropriate eating behavior and/or being overweight/obese and/or non-communicable chronic diseases. We will provide a questionnaire (33) and guidelines (https://vamos.ufsc.br/orientacoes-para-questionario-vamos/) to PHC professionals to screen participants. This screening will be carried out through a meeting scheduled prior to the start of the program with interested users. At this meeting, in addition to screening assessments, PHC professionals will be recommended to clarify the objectives and methodology of the program. In addition, we will assess the reasons for participants' absences and dropouts throughout the program. For this, we use the PHC professionals' monitoring records (attendance list and replacement of the sections), which will be guided to record the date and reason for the termination of each participant.

**Effectiveness**, assessed at the individual level, refers to the extent of the intervention's impact on the primary outcomes, how it changes behavior and affects the quality of life, and whether or not there are unintended results (positive or negative) (22). In this study, effectiveness will be assessed considering the primary outcomes of BCP-VAMOS: physical activity and eating behavior. Physical activity will be assessed through a self-report of the weekly practice (type, frequency, and duration) (34) and by accelerometry (35), which allows measuring the time spent on sedentary behavior, light, moderate, and vigorous physical activity. Eating behavior will be assessed through the habit and frequency of consumption of foods classified as healthy (fruits, greens, and raw or cooked vegetables) and unhealthy (sweets, snacks, and sugary drinks) (34). These data will be collected by the PHC professionals through a validated questionnaire for the BMC-VAMOS program (33). The effectiveness outcomes will be analyzed by PHC professional (staff level) to assess the impact of intervention strategies, comparing the IG to the CG.

**Adoption** is a measure at the organizational level of participation that refers to the representativeness of locations and/or agents who are willing to implement an intervention, considering those who are eligible (22). In this study, the number, proportion, and representativeness of local/PHC professionals who initiate the program delivery will be calculated using those certified by the online training with eligibility criteria. We will calculated the adoption rate by considering the ratio between the number of PHC professionals who initiated the implementation of the program in each UBS and the number of PHC professionals certified by the online training, multiplied by 100. To identify the factors that will influence the program adoption and non-adoption, qualitative data will be collected through interviews with PHC professionals.

**Implementation** at the organizational level refers to the degree to which delivery agents comply with several elements of an intervention protocol (22). In this study, the quality of the BCP-VAMOS implementation will be assessed based on strategies for recruiting, retaining, and monitoring participants. This analysis will be performed by PHC professional, comparing the IG to the CG. During a 1-year follow-up, a detailed manual will be developed, containing all the implemented strategies and the approximate dates for the completion of each intervention component. The manual will help in the development of checklists to assess the fidelity of the intervention delivery and the degree to which strategies are implemented as intended. A trained researcher will visit the intervention sites to observe and complete the checklist, in addition to conducting interviews with PHC professionals.

**Maintenance** at the organizational level assesses the extent to which an intervention becomes institutionalized or part of routine practices and organizational policies (22). In this study, the assessment of maintenance aims to show if new groups will be delivered during the implementation process of the BCP-VAMOS or if the local organization is interested in institutionalizing the program. This evaluation will be carried out shortly after the last section of the program, through an interview with PHC professionals and managers.

The CFIR pragmatic framework (23), widely used in implementation research in the health area (36), will be used to qualify the outcomes measures. CFIR describes 37 implementation constructs, categorized into five domains: innovation/intervention characteristics, outer setting, inner setting, characteristics of the individuals involved, and process of implementation (23).

**Innovation characteristics** directly influence the success of the implementation (36). The constructs evaluated in this domain are related to information about the *source of the intervention, quality of evidence, organizational advantage, adaptability, trialability, complexity, design and presentation of the intervention, and costs* (23). In this study, we are especially interested in adaptability—the degree to which the intervention can be customized or refined to meet local needs.

Increasingly recognized as an active part of implementation, the **outer setting** domain is composed of constructs that address *patient needs and resources, cosmopolitanism, peer pressure, external policies, and incentives* (23). Health systems are hierarchically organized and interconnected. Changes in the external environment can influence, positively or negatively, implementation (23). Identifying these factors in this study may help other organizations to replicate implementation successfully.

The **inner setting** at the organizational level describes constructs that relate *structural characteristics, networks and communications, culture, implementation climate, and readiness for implementation*. These aspects are interrelated and influence implementation (23). In this study, we will evaluate the quality of internal communication, adaptability in decision-making, receptivity of the individuals involved, and organizational commitment to intervention.

Organizations are composed of individuals and their behaviors. Little is known about the interaction between individuals and their propagating effects through teams and organizations in implementation (36). The domain regarding **characteristics of individuals** lists constructs that can reveal *knowledge and beliefs of the professionals involved, self-efficacy for implementation, personal stage of change, identification with the organization*, and *other personal attributes and values* (23). All these items can influence the degree of commitment to service innovation and affect the implementation of an intervention (36). Trying to understand these influences is part of this study, as they can further qualify the health professionals' training for the implementation of the program on future occasions.

**Process** is the domain that represents the greatest challenge during program evaluations in public health (23, 36). It is evaluated through the constructs of *planning, engagement, execution*, and *reflection, and evaluation of the professionals involved in delivering the intervention*. The better each of these four mechanisms is implemented, the better and more effective the implementation will be (23). Thus, in this study, all items will be evaluated.

CFIR will be used to gather information about the constructs that affect the implementation of the innovation. It will also aid in the identification of the contextual determinants (barriers and facilitators) that can be used to guide the selection of the most appropriate strategies for the success of the intervention (see Table 2). These data will be collected through interviews based on the CFIR matrix (www.cfirguide.org). From the perception of PHC professionals, managers and participants of the PMC-VAMOS, we will classify and code the barriers and facilitators within each of the domains and constructs of the CFIR.

### Data collection

The BCP-VAMOS implementation process will be monitored for 12 months. The data will be collected through electronic forms, a smartphone messaging application, observations, and semi-structured interviews with key informants involved in the implementation strategies. The interviews will be conducted by phone or in-person by an independent co-investigator who will not participate in the other stages of the study. The variables referring to BCP-VAMOS participants will be collected by the health professionals responsible for implementation at the baseline and during post-intervention. This information will be recorded and shared with the research team using electronic spreadsheets. A trained researcher will collect accelerometry data *in loco*. The instruments for collecting data in each stage of the study will be developed based on recent recommendations for pragmatic studies (28), the RE-AIM checklist (37), the CFIR matrices (www.cfirguide.org), and the questionnaire for effectiveness evaluation, designed for the BCP-VAMOS (33). Participants' consent will be collected by health professionals before the intervention starts. We will train independent research to evaluate the process. All data regarding the implementation of BCP-VAMOS will be managed by one single researcher to maintain data quality and reliability. The confidentiality of the participant is guaranteed using identification code numbers to match the treatment data in the computer files for each group.

### Data analysis

Quantitative data will be expressed through descriptive analysis (absolute and relative frequency, mean and standard deviation) and inferential analysis to compare the impact of the intervention between the groups. To compare the characteristics between the groups, Fischer's exact and chi-square tests will be used. To verify the effect of the intervention, Student's paired t test or Wilcoxon test (continuous variables) and McNemar test (categorical variables) will be used. To compare changes between groups, Student's t test or Mann-Whitney *U*-test will be applied. The analyses will be performed using a 5% probability, and will be generated using the Statistical Package for the Social Sciences (SPSS)^®^ software, version 22.0. Qualitative data will be fully transcribed by a researcher, and their quality will be assessed by a second researcher. Through the technique of descriptive content analysis (38), the texts will be pre-analyzed (each interview will be read), coded (reduced to units of meaning consistent with the purpose of the study), and categorized (the participants' answers will be organized). The information will be coded deductively, following the conceptual model of the study and, if necessary, inductive codes will be added until saturation is achieved (39). The data will be double-coded in the QSR NVivo^®^ 12.0 software by two trained researchers. Intercoder reliability will be assessed employing Cohen's kappa (criterion> 0.80).

## Discussions

Implementation Science has emerged as a field of investigation to translate EBPs into community settings (2), and examine the most promising way to disseminate, implement and sustain interventions in healthcare settings (9, 14). However, the success of the implementation depends not only on the health professionals' learning about the intervention itself but also on their ability to overcome barriers to implementation. This is particularly important for complex skills that cannot be fully taught in traditional training (12, 14).

Through ongoing support, it is possible to provide more information for health professionals and enhance the solution of problems through a variety of resources, right when they are needed and at all levels—intervention, organization, provider, and participant (15). This includes planning, which can generate adaptations in practice and influence successful refinements (14, 15).

Action planning is a strategy that involves setting goals, identifying obstacles, and creating strategies to overcome them. This method is often used in behavioral interventions and public health practices and has shown positive results (16). When it focuses on the provider, action planning can be used to provide additional structure (16), thus, it can be a tool to assist in the process of implementing a health innovation.

This study has the potential for increasing success in the implementation of an intervention whose purpose is to qualify integrated care in an organizational health system. Implementing interventions brings together components of organizational interaction. It may require new attitudes by the individuals involved, along with showing diversity in the results that influence the context (40). Moreover, effective knowledge translation optimizes access to protocols that aim to improve public health policies (8). Diligent protocol planning is critical so that future interventions have a better chance of being effective when evaluated and adopted in the real world (19).

The literature provides an extensive list of D&I models to assist in the process. These models, aim to plan, monitor, and evaluate the implementation of interventions (5). The combined use of them has been recommended to determine whether an intervention is pragmatic and generalizable, and, if so, to what extent (24–26). In our protocol, we propose the associated use of RE-AIM (22) and CFIR (23) in an attempt to approach the greatest number of items related to success in the implementation of a behavior change intervention. These models deal with factors that can impact implementation processes and outcomes. Combined use may provide, in addition to specific aspects that must be assessed, the various levels of influence on implementation. Thus, in addition to helping to guide implementation practice, they are potentially useful for designing and executing implementation strategies that aim to change relevant determinants, such as changing the behavior of PHC professionals or adherence to a clinical guidelines (6).

RE-AIM can be used to systematically capture perceptions, decision making and impacts on population and “real life” scenarios (24, 25). This is essential for understanding why and how interventions are adopted, implemented, and maintained in public health organizations (18). CFIR provides a framework for approaching complex and iterative states, understanding the dynamic, multi-level and transient nature of implementation in pragmatic settings. Its main objective is to consolidate findings about the reasons (barriers and enablers) why implementations may fail or succeed (22). Thus, the CFIR will be able to contribute to the assessment of adaptation, sustainability and impact outcomes, aiding the investigation factors that influence implementation and how they affect the development of the intervention (41).

Therefore, it is possible to consider the combined use of these two frameworks to understand what will or will not work in the implementation of BCP-VAMOS. This information can be used to plan activities to improve the quality and sustainability of the program in the long term. Characterized as a health innovation, BCP-VAMOS combines evidence-based behavioral strategies in a protocol that aims to motivate adults and elderly in the community to adopt an active and healthy lifestyle (20). The online training for implementing the program was created in a virtual format based on self-instructional teaching methods (21). Besides facilitating adoption, the online training includes detailed descriptions of the program's structure and content. Additionally, it offers experiential learning opportunities that can be used to manage and conduct the intervention and other health promotion activities.

On the other hand, we identified the difficulty of recruiting PHC professionals in different places, particularly in health centers. The workload reported by PHC professionals also influences the rate of adoption and success in implementing community-based behavior change interventions. However, to minimize these aspects, the health managers should encourage the PHC professionals to implement programs that directly involved the population. In addition, use local media to reach the community in these interventions.

## Conclusion

As far as we know, this is the first study in Brazil that uses a digital platform to disseminate and implement a large-scale community-based intervention in public health. An PRCT with a mixed-methods approach, comparing the success of implementation between groups with and without ongoing support, is also unprecedented when it comes to a behavior change program for physical activity and healthy eating in PHC, particularly in the Brazilian context. For the developing team of BCP-VAMOS, this study will provide information about the success or failure of the implementation, program fidelity, and the need to adapt the intervention in the real world.

Thus, we propose that the set of information and findings will provide practical guidance for researchers and policy-makers in the area of health, allowing the use of evidence-based approaches to develop similar protocols to implement and adapt public health interventions. Finally, we believe that the development of interventions in the real world is an opportunity to create a positive impact on the applicability and sustainability of future practices, and can contribute to new evidence in Behavioral Science and Implementation Science.

## Data availability statement

The datasets presented in this study can be found in online repositories. The names of the repository/repositories and accession number(s) can be found at: Brazilian Registry of Clinical Trials (http://www.ensaiosclinicos.gov.br/) by the RBR-2VW77Q indicator.

## Ethics statement

The studies involving human participants were reviewed and approved by Ethics Committee of the Federal University of Santa Catarina (n. 1.394.492). The patients/participants provided their written informed consent to participate in this study.

## Author contributions

TB is the Chief Investigator and contributed to the supervision and writing-original draft preparation. LK, FB, and FA contributed to the conception and design of the study. CR, EM, and CT contributed to the writing-reviewing and editing. All authors contributed to manuscript revision, read, and approved the submitted version.

## Funding

This research was supported by the Fundação de Amparo à Pesquisa e Inovação do Estado de Santa Catarina (FAPESC) through the Programa Pesquisa para o SUS (PPSUS, N2016TR2210, Grant 484/2016) and by the Coordenação de Aperfeiçoamento de Pessoal de Nível Superior (CAPES), Brazil (Finance Code 001).

## Conflict of interest

The authors declare that the research was conducted in the absence of any commercial or financial relationships that could be construed as a potential conflict of interest.

## Publisher's note

All claims expressed in this article are solely those of the authors and do not necessarily represent those of their affiliated organizations, or those of the publisher, the editors and the reviewers. Any product that may be evaluated in this article, or claim that may be made by its manufacturer, is not guaranteed or endorsed by the publisher.

## Abbreviations

BCP-VAMOS: Active Life Improving Health-Behavior Change Program
CI: Implementation Science
CCT + APA: Consultee-Centered Training + Action Planning Approach
CFIR: Consolidated Framework for Implementation Research
D&I: Dissemination and Implementation
EBP: Evidence-Based Practice
PHC: Primary Health Care
RE-AIM: Reach, Effectiveness, Adoption, Implementation, Maintenance
PRCT: Pragmatic Randomized Controlled Trial
TDA: Traditional Didactic Approach.

## References

[1] World Health Organization (WHO). Global Strategy on Diet, Physical Activity, and Health. Geneva: WHO (2004).

[2] Escoffery C Lebow-Skelley E Haarrdoerfer R Boing E Udelson H Wood R . A systematic review of adaptations of evidence-based public health interventions globally. Implement Sci. (2018) 13:125. 10.1186/s13012-018-0815-9 30257683 PMC6158804

[3] Estabrooks PA Brownson RC Pronk NP. Dissemination and implementation science for public health professionals: an overview and call to action. Prev Chronic Dis. (2018) 15:E162. 10.5888/pcd15.180525 30576272 PMC6307829

[4] Powell BJ Waltz TJ Chinman MJ Damschroder LJ Smith JL Matthieu MM . A refined compilation of implementation strategies: results from the Expert Recommendations for Implementing Change (ERIC) project. Implement Sci. (2015) 10:21. 10.1186/s13012-015-0209-1 25889199 PMC4328074

[5] Tabak RG Khoong EC Chambers DA Brownson RC. Bridging research and practice: models for dissemination and implementation research. Am J Prev Med. (2012) 43:337–50. 10.1016/j.amepre.2012.05.024 22898128 PMC3592983

[6] Nilsen P. Making sense of implementation theories, models, and frameworks. Implement Sci. (2015) 10:53. 10.1186/s13012-015-0242-0 25895742 PMC4406164

[7] Chambers DA Norton WE. The adaptome. Advancing the science of intervention adaptation. Am J Prev Med. (2016) 51:S124–31. 10.1016/j.amepre.2016.05.011 27371105 PMC5030159

[8] Malla C Aylward P Ward P. Knowledge translation for public health in low and middle-income countries: a critical interpretive synthesis. Glob Health Res Policy. (2018) 3:29. 10.1186/s41256-018-0084-9 30377666 PMC6196454

[9] Lewis CC Klasnja P Powell BJ Lyon AR Tuzzio L Jones S . From classification to causality: advancing understanding of mechanisms of change in implementation science. Front. Public Health. (2018) 6:136. 10.3389/fpubh.2018.00136 29868544 PMC5949843

[10] Kitson AL Harvey G. Methods to succeed in effective knowledge translation in clinical practice. J Nurs Scholarship. (2016) 48:294–302. 10.1111/jnu.12206 27074390

[11] Herschell AD Kolko DJ Baumann BL Davis AC. The role of therapist training in the implementation of psychosocial treatments: a review and critique with recommendations. Clin Psychol Rev. (2010) 30:448–66. 10.1016/j.cpr.2010.02.005 20304542 PMC2872187

[12] Lau R Stevenson F Ong BN Krysia D Treweek S Eldridge S . Achieving change in primary care-effectiveness of strategies for improving implementation of complex interventions: systematic review of reviews. BMJ Open. (2015) 5:e009993. 10.1136/bmjopen-2015-009993 26700290 PMC4691771

[13] Beidas RS Edmunds JM Marcus SC Kendal PC. Training and consultation to promote implementation of an empirically supported treatment: a randomized trial. Psychiatr Ser. (2012) 63:660–5. 10.1176/appi.ps.201100401 22549401 PMC3432154

[14] Edmunds JM Beidas RS Kendall PC. Dissemination and implementation of evidence-based practice: training and consultation as implementation strategies. Clin Psychol. (2013) 1–20:152–65. 10.1111/cpsp.12031 24072959 PMC3780425

[15] Nadeem E Gleacher A Beidas RS. Consultation as an implementation strategy for evidence-based practices across multiple contexts: unpacking the black box. Adm Policy Ment Health. (2013) 40:439–50. 10.1007/s10488-013-0502-8 23716145 PMC3795855

[16] Hagger MS Luszczynska A. Implementation intention and action planning interventions in health contexts: state of the research and proposals for the way forward. Appl Psychol Health Well Being. (2014) 6:1–47. 10.1111/aphw.12017 24591064

[17] Huynh AK Hamilton AB Farmer MM Bean-Mayberry B Stirman SW Moin T . A pragmatic approach to guide implementation evaluation research: strategy mapping for complex interventions. Front Public Health. (2018) 6:134. 10.3389/fpubh.2018.00134 29868542 PMC5968102

[18] Glasgow RE Estabrooks PE. Peer-reviewed: pragmatic applications of RE-AIM for health care initiatives in community and clinical settings. Prevent Chron Dis. (2018) 15:170271. 10.5888/pcd15.170271 29300695 PMC5757385

[19] O'Cathain A Croot L Duncan E Rousseau N Sworn K Turner KM . Guidance on how to develop complex interventions to improve health and healthcare. BMJ Open. (2019) 9:e029954. 10.1136/bmjopen-2019-029954 31420394 PMC6701588

[20] Benedetti TRB Manta SW Gomez LSR Rech CR. Logical model of a behavior change program for community intervention - Active Life Improving Health - VAMOS. Rev Bras Ativ Fí*s Saúde*. (2017) 22:309–13. 10.12820/rbafs.v.22n3p309-313

[21] Konrad LM Ribeiro CG Tomicki C Benedetti TRB. Validation of educational technology to implement a community program in public health. Rev Bras Ativ Fí*s Saúde*. (2020) 25:e0155. 10.12820/rbafs.25e0155

[22] Glasgow R Vogt T Boles S. Evaluating the public health impact of health promotion interventions: the RE-AIM framework. Am J Public Health. (1999) 9:1322–7. 10.2105/AJPH.89.9.1322 10474547 PMC1508772

[23] Damschroder LJ Aron DC Keith RE Kirsh SR Alexander JA Lowery JC. Fostering implementation of health services research findings into practice: a consolidated framework for advancing implementation science. Implement Sci. (2009) 4:50. 10.1186/1748-5908-4-50 19664226 PMC2736161

[24] Glasgow RE Harden SM Gaglio B Rabin B Smith ML Porter GC . RE-AIM planning and evaluation framework: adapting to new science and practice with a 20-year review. Front Public Health. (2019) 7:64. 10.3389/fpubh.2019.00064 30984733 PMC6450067

[25] Kwan BM McGinnes HL Ory MG Estabrooks PA Waxmonsky JA Glasgow RE. RE-AIM in the real world: use of the RE-AIM framework for program planning and evaluation in clinical and community settings. Front Public Health. (2019) 7:345. 10.3389/fpubh.2019.00345 31824911 PMC6883916

[26] King DK Shoup JA Raebel MA Anderson CB Wagner NM Ritzwoller DP . Planning for implementation success using RE-AIM and CFIR frameworks: a qualitative study. Front Public Health. (2020) 8:59. 10.3389/fpubh.2020.00059 32195217 PMC7063029

[27] Loudon K Treweek S Sullivan F Donnan P Thorpe KE Zwarenstein M. The PRECIS-2 tool: designing trials that are fit for purpose. BMJ. (2015) 350:h2147. 10.1136/bmj.h2147 25956159

[28] Chan AW Tetzlaff JM Altman DG Laupacis A Gøtzsche PC KrleŽa-Jerić K . SPIRIT 2013 statement: defining standard protocol items for clinical trials. Ann Intern Med. (2013) 158:200–7. 10.7326/0003-4819-158-3-201302050-00583 23295957 PMC5114123

[29] Ribeiro CG Konrad LM Tomicki C Almeida FA Brito FA Benedetti TRB. Evaluation of the didactic material of the “Active Life Improving Health” Program (VAMOS), version 3.0. e-Revista LOGO. (2021) 10:71–92. 10.26771/e-Revista.LOGO/2021.1.04 34211743 PMC8200621

[30] Brazil. Unified Health System Data Department. DATASUS. (2019). Available online at: http://tabnet.datasus.gov.br (accessed January 10, 2019).

[31] Perry CK Damschroder LJ Hemler JR Woodson TT Ono SS Cohen DJ. Specifying and comparing implementation strategies across seven large implementation interventions: a practical application of theory. Implement Sci. (2019) 14:32. 10.1186/s13012-019-0876-4 30898133 PMC6429753

[32] Proctor EK Powell BJ McMillen JC. Implementation strategies: recommendations for specifying and reporting. Implement Sci. (2013) 8:139. 10.1186/1748-5908-8-139 24289295 PMC3882890

[33] Silva MC Ribeiro CG Benedetti TR. VAMOS program: instruments for measuring physical activity, feeding, and anthropometry. Rev Bras Cineantropom Desempenho Hum. (2020) 22:e58256. 10.1590/1980-0037.2020v22e58256

[34] Brazil. Ministry of Health. Health Surveillance Secretary. VIGITEL Brasil 2016: Surveillance of Risk and Protective Factors for Chronic Diseases by Telephone Survey: Estimates on the Frequency and Sociodemographic Distribution of Risk and Protective Factors for Chronic Diseases in the Capitals of the 26 Brazilian States and the Federal District in 2016. Brasília: Ministry of Health (2017). Available online at: https://portalarquivos2.saude.gov.br/images/pdf/2018/marco/02/vigitel-brasil-2016.pdf (accessed march 20, 2019).

[35] Sasaki JE Silva KS Da Costa BGG. Using an Accelerometer to Measure Physical Activity And Sedentary Behavior: What Do We Need to Know? 1st ed. Londrina: Midiograf (2018).

[36] Means AR Kemp CG Gwayi-Chore MC Gimbel S Soi C Sherr K . Evaluating and optimizing the Consolidated Framework for Implementation Research (CFIR) for use in low- and middle-income countries: a systematic review. Implement Sci. (2020) 15:17. 10.1186/s13012-020-0977-0 32164692 PMC7069199

[37] Brito FA Benedetti TRB Tomicki C Konrad LM Sandreschi P Manta SW . Translation and adaptation of the RE-AIM check list for Brazilian reality. Rev Bras Ativ Fí*s Saúde*. (2018) 23e0033. 10.12820/rbafs.23e0033

[38] Hsieh H-F Shannon SE. Three approaches to qualitative content analysis. Qual Health Res. (2005) 15:1277–88. 10.1177/1049732305276687 16204405

[39] Malterud K Siersma V Guassora A. Sample size in qualitative interview studies: guided by information power. Qual Health Res. (2016) 26:1753–60. 10.1177/1049732315617444 26613970

[40] Craig P Ruggiero ED Frohlich KL Mykhalovskyv E White M. Taking Account of Context in Population Health Intervention Research: Guidance for Producer, Users, and Funders of Research. Southampton: NIHR Evaluation, Trials and Studies Coordinating Centre (2018).

[41] Kirk AM Kelley C Yankeys N Birken SA Abadie B Damschroder L. A systematic review of the use of the Consolidated Framework for Implementation Research. Implement Sci. (2016) 11:72. 10.1186/s13012-016-0437-z 27189233 PMC4869309

